# The first complete chloroplast genome sequence of an early spring flowering plant, *Corydalis repens*

**DOI:** 10.1080/23802359.2025.2603836

**Published:** 2026-02-19

**Authors:** Huanchu Liu, Xingyuan He, Wei Chen, Yanqing Huang, Yang Liu

**Affiliations:** a Key Laboratory of Forest Ecology and Management, Institute of Applied Ecology, Chinese Academy of Sciences, Shenyang, China; b Shenyang Arboretum, Chinese Academy of Sciences, Shenyang, China; c Liaoning Shenyang Urban Ecosystem Research Station, National Forestry and Grassland Administration, Shenyang, China; d Dandong Forestry and Grassland Development Service Center, Dandong, China

**Keywords:** *Corydalis*, chloroplast genome, phylogenetic analysis

## Abstract

*Corydalis repens* Mandl & Muhldorf is an early spring-blooming species within the genus *Corydalis*. In this study, we present the complete chloroplast genome of *C. repens* for the first time. The assembled genome is 188,101 base pairs (bp) long and is organized into a typical quadripartite structure consisting of a large single-copy (LSC) region of 89,844 bp, a small single-copy (SSC) region of 757 bp, and two inverted repeat (IR) regions totaling 48,750 bp. The overall GC content is 40.2%. The plastome encodes 141 genes, including 94 protein-coding genes, 38 transfer RNA genes, and eight ribosomal RNA genes, along with a single pseudogene (*clpP*). Phylogenetic reconstruction indicates that *C. repens* is nested within section *Corydalis* and shows the closest affinity to *C. maculata*. These findings provide an important genetic resource for future studies on the evolutionary history and conservation of the genus *Corydalis*.

## 1. Introduction

*Corydalis* DC., one of the largest genera within the Papaveraceae, includes roughly 500 recognized species (Liu et al. 2024). Members of this genus are broadly distributed throughout the Northern Hemisphere, with their greatest richness concentrated in the Himalayan–Hengduan Mountains region (Wang 2006; Chen et al. 2023). Owing to its extensive morphological diversity across varied environments, *Corydalis* has long been regarded as one of the most taxonomically difficult groups to classify (Ren et al. 2019; Kim et al. 2023). Plastid (chloroplast) genome characterized by conserved structure, uniparental inheritance, and the absence of recombination—serve as powerful markers for phylogenetic inference in plants (Ravi et al. 2008) and are increasingly used for species identification and lineage delimitation (Yang et al. 2013). In *Corydalis*, where rapid radiation and recurrent morphological convergence often obscure taxonomic boundaries, plastome data offer a reliable molecular basis for reconstructing evolutionary patterns. Although plastid genomes from a growing number of *Corydalis* species have been sequenced, a substantial portion of this large genus still lacks genomic characterization.

*Corydalis repens* Mandl & Muhldorf, a representative species of section *Corydalis*, is characterized by its pale blue to bluish-purple or reddish-purple flowers. It can be readily distinguished from closely related taxa by its foliage, which frequently bears whitish streaks or spots and may exhibit either smooth margins or margins with coarse papillae (Wu et al. 1999). The tubers of *C. repens* are known to contain several pharmacologically active alkaloids, including protopine and corydaline, both of which have documented medicinal significance (Ren et al. 2020). In this work, we generated the complete chloroplast genome of *C. repens* to examine its structural organization and genomic features. The resulting plastome data provide valuable resources for species delimitation, evolutionary analyses, and conservation strategies within this taxonomically challenging genus.

## 2. Materials and methods

### 2.1. Sampling

**Figure 1.**
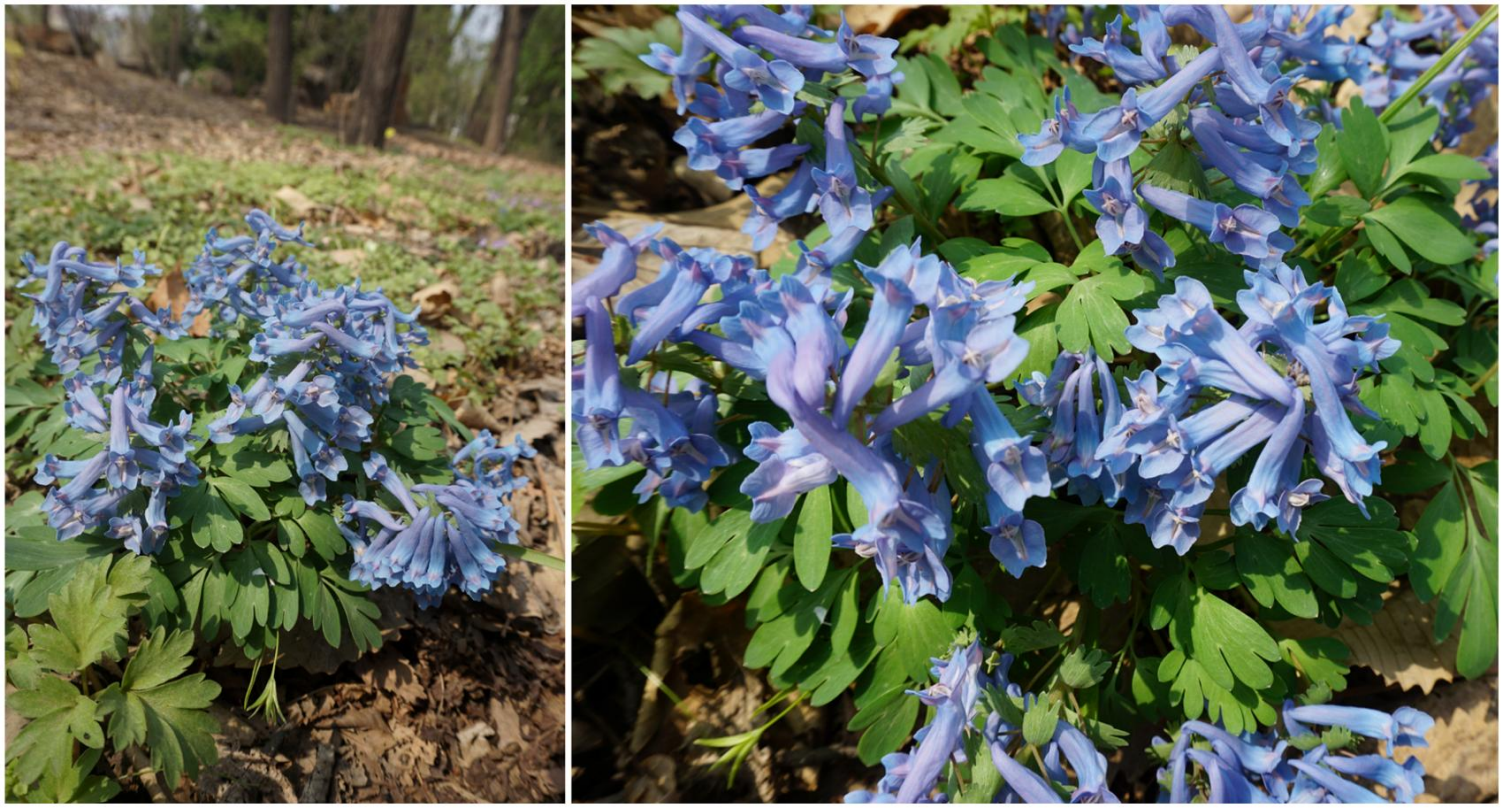
*Corydalis repens*. The photos were taken by Huanchu Liu in the Arboretum (Shenyang Arboretum, Chinese Academy of Sciences). The leaves exhibit pale whitish stripes. The flowers are bluish-purple, with broadly spreading outer petals and a slightly notched apex.

A young leaf of *C. repens* was collected in Shenyang, Liaoning Province, China (41.7769°N, 123.4627°E) by Huanchu Liu (Figure 1) and immediately dried in silica gel. The specimen was taxonomically verified by Yanqing Huang and deposited in the Herbarium of Northeast China, Institute of Applied Ecology, Chinese Academy of Sciences, under the voucher number IFP0260036. For further inquiries, the curator can be contacted at huangyanqing@iae.ac.cn.

### 2.2. DNA extraction and sequencing

Genomic DNA was isolated from silica gel-dried leaves following a modified CTAB extraction protocol (Doyle and Doyle 1987). A sequencing library with an average insert size of 150 bp was prepared using the NovaSeq Xplus DNA Library Preparation Kit (Illumina Inc., San Diego, CA, USA). Raw reads were subsequently quality-filtered using fastp v0.23.2 to remove low-quality sequences (Chen et al. 2023).

### 2.3. Assembly and annotation

The filtered reads were assembled into the complete chloroplast genome using GetOrganelle (Jin et al. 2020), with the *Corydalis remota* chloroplast genome (accession number NC_072183) as a reference. Gene annotation was performed with the online tool GeSeq (Tillich et al. 2017) using default settings to identify protein-coding genes, transfer RNA (tRNA) genes, and ribosomal RNA (rRNA) genes. A circular map of the *C. repens* chloroplast genome was generated using Chloroplot (Zheng et al. 2020), while intron–exon structures and schematic representations of cis- and trans-splicing genes were visualized using CPGView (Liu et al. 2023). The complete chloroplast genome of *C. repens* was successfully assembled and has been deposited in GenBank under accession number PV658157.

### 2.4. Phylogenetic analysis

Sequence alignments were performed using MAFFT (Katoh and Standley 2013). A total of 26 representative *Corydalis* species, with an emphasis on those from section *Corydalis*, were included in the analysis, while *Fumaria officinalis* (Papaveraceae) was designated as the outgroup. Maximum likelihood (ML) phylogenetic reconstruction was carried out in RAxML v8.2.12 using the GTR + Γ (GTRGAMMA) nucleotide substitution model, with 1,000 rapid bootstrap replicates to assess branch support. The resulting phylogenetic tree was visualized using the online tool Chiplot (Xie et al. 2023).

## 3. Results

**Figure 2.**
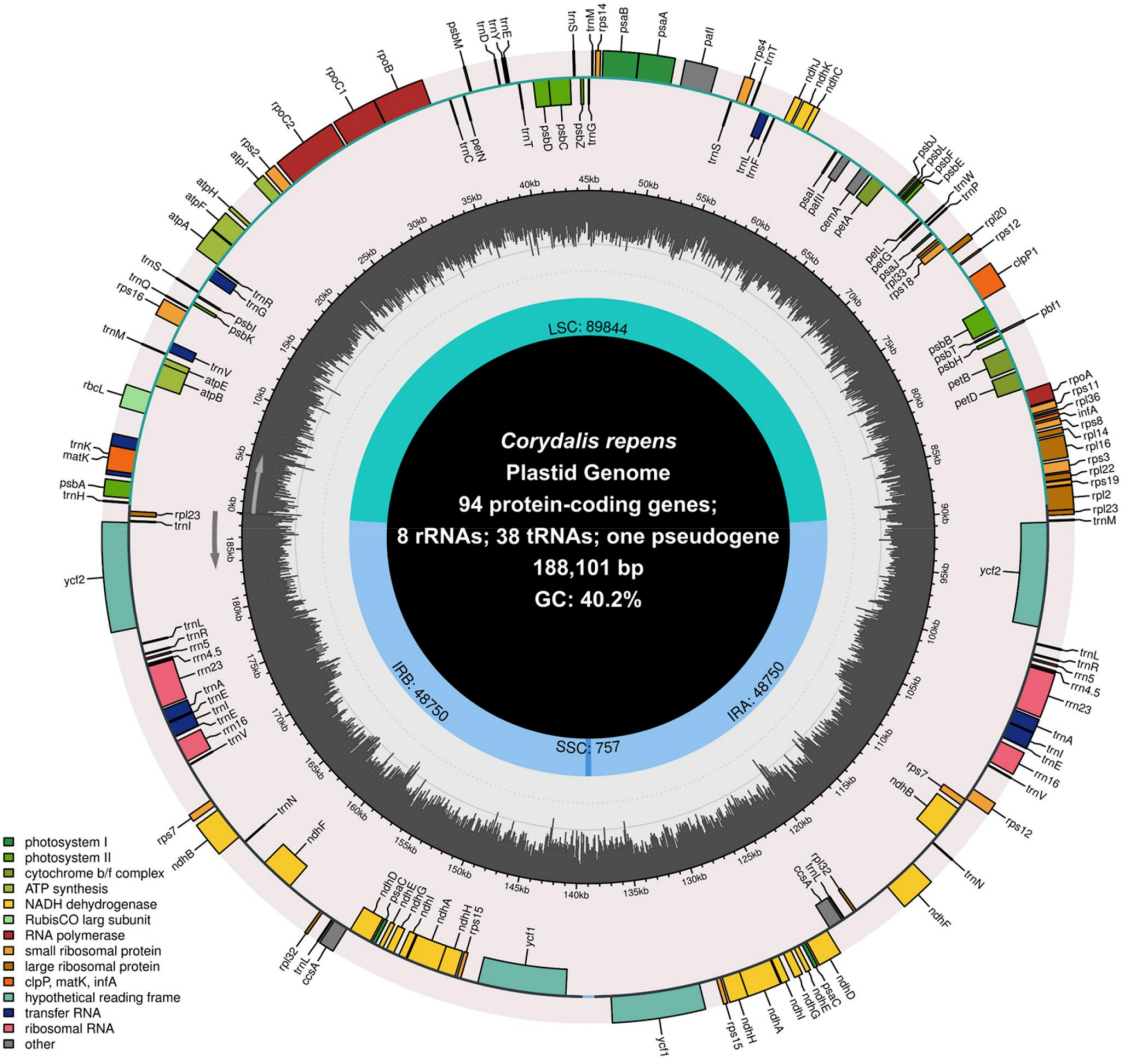
Circular map of the *C. repens* chloroplast genome. Genes with different functions are shown in different colors. Genes within circles are transcribed clockwise and genes outside circles are transcribed counterclockwise. The darker grey in the inner circle represent GC content. LSC, large single-copy region; SSC, small single-copy region; IR, inverted repeat.

To verify the accuracy of the genome assembly, coverage depth analysis was performed, yielding an average depth of 9,333.89, with a maximum of 370,562 and a minimum of 53 (Figure S1). The complete chloroplast genome of *C. repens* is 188,101 bp in length and displays a typical quadripartite structure, comprising a large single-copy (LSC) region of 89,844 bp, a small single-copy (SSC) region of 757 bp, and a pair of inverted repeat (IR) regions totaling 48,750 bp (Figure 2). The overall GC content is 40.2%. A total of 141 genes were annotated, including 94 protein-coding genes, 38 tRNA genes, and 8 rRNA genes, along with one pseudogene (*clpP*). Within the IR regions, four rRNA genes (*rrn16, rrn23, rrn4.5, and rrn5*), eight tRNA genes (*trnA-UGC, trnA-UUC, trnL-CAA, trnL-UAG, trnM-CAU, trnN-GUU, trnR*-*ACG*, and *trnV-GAC*), and 18 protein-coding genes are duplicated. Intron–exon structure analysis revealed that two protein-coding genes, *rps12* and *pafI*, each contain two introns. Schematic representations of cis- and trans-splicing genes are provided in Supplemental Figures S2 and S3.

**Figure 3.**
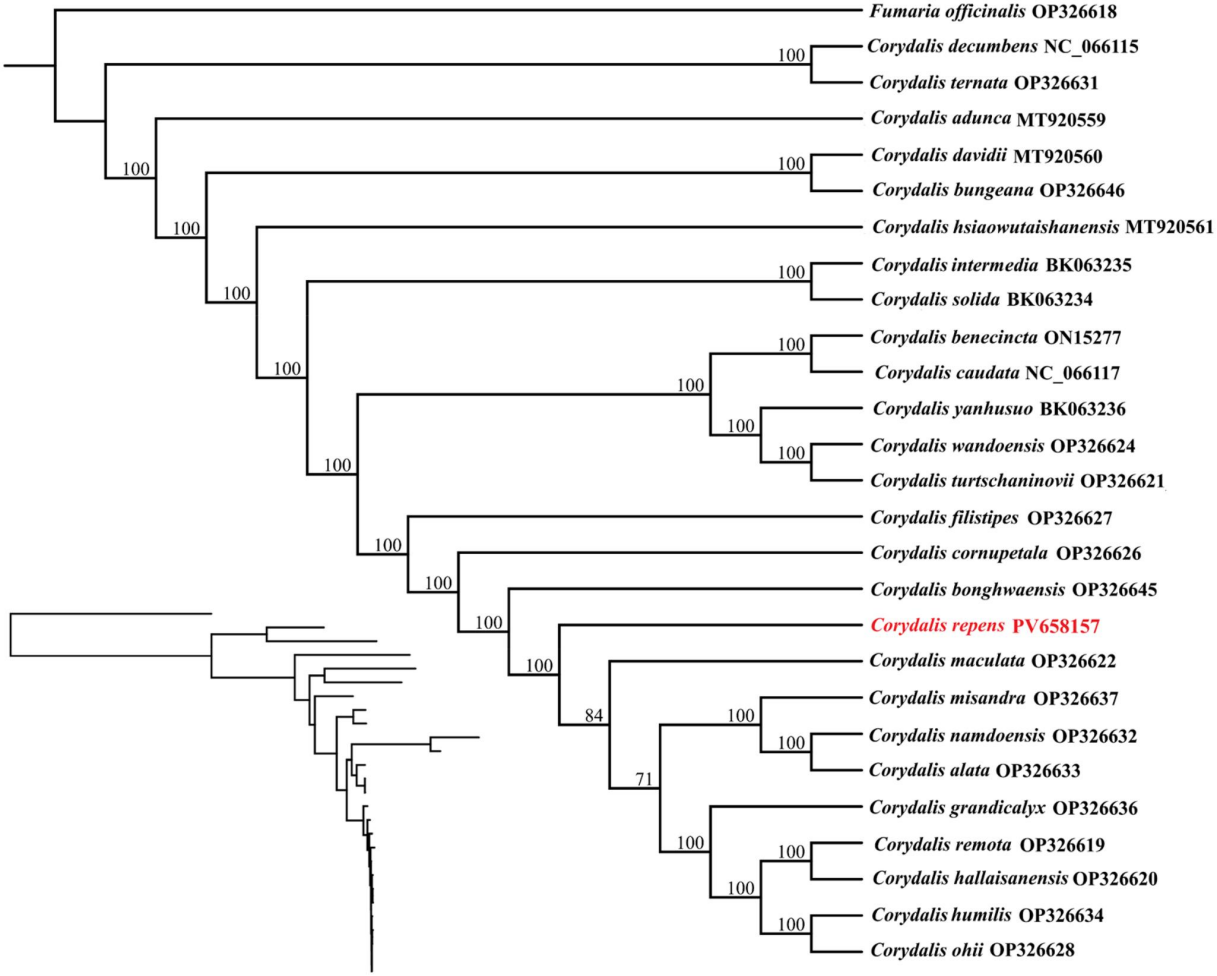
Phylogenetic tree using maximum-likelihood (ML) based on chloroplast genomes of 26 *Corydalis* species with *Fumaria officinalis* as an outgroup. Numbers above nodes are support values with ML bootstrap (BS) values. The sequences used for constructing the phylogenetic tree are as follows: *Fumaria officinalis* (OP326618), *C. bungeana* (OP326646), *C. intermedia* (BK063235), *C. solida* (BK063234), *C. filistipes* (OP326627), *C. cornupetala* (OP326626), *C. maculata* (OP326622), *C. namdoensis* (OP326632), *C. lata* (OP326633), *C. misandra* (OP326637), *C. grandicalyx* (OP326636), *C. remota* (OP326619), *C. hallaisanensis* (OP326620), *C. humilis* (OP326634), *C. ohii* (OP326628), *C. bonghwaensis* (OP326645), *C. wandoensis* OP326624, *C. turtschaninovii* (OP326621), *C. yanhusuo* (BK063236), *C. ternata* (OP326631)(Kim et al. 2023); *C. dunca* (MT920559), *C. davidii* (MT920560), *C. hsiaowutaishanensis* (MT920561) (Xu et al. 2021); *C. benecincta* (ON152778), *C. caudata* (NC_066117), *C. decumbens* (NC_066115) (Xu et al. 2022). The chloroplast genomes of *Corydalis repens* in this study were highlighted in red color.

The phylogenetic tree reconstructed using 27 complete chloroplast genomes clarifies the placement of *C. repens* within the genus *Corydalis* (Figure 3). In this analysis, *C. repens* clusters closely with species from the subgenus *Corydalis* and section *Corydalis*, consistent with previous studies (Chen et al. 2023; Kim et al. 2023). Most branches are strongly supported, with bootstrap values of 100, indicating a high level of phylogenetic resolution. Our results indicate that *C. repens* forms a sister clade with the common ancestor of *C. maculata*, *C. misandra*, *C. namdoensis*, *C. alata*, *C. grandicalyx*, *C. remota*, *C. hallaisanensis*, *C. humilis*, and *C. ohii*. Quartet statistics for 26 *Corydalis* species also reveal substantial variation in the lengths of the SSC (71–14,845 bp) and IR (26,808–52,755 bp) regions (Table S1).

## 4. Discussion and conclusion

This study provides the first complete characterization of the chloroplast genome of *C. repens*, which contains 141 genes, including 94 protein-coding genes, 38 tRNA genes, and 8 rRNA genes. The plastome of *C. repens* exhibits structural features similar to those of closely related species such as *C. maculata*, *C. misandra*, and *C. namdoensis*, suggesting that chloroplast genomes within section *Corydalis* are relatively conserved during evolution. Phylogenetic analysis further clarified the position of *C. repens* within the genus, revealing a close relationship with *C. maculata*, in agreement with previous studies. Notably, the IR region in *C. repens* extends into *ycf1*, while the SSC region is markedly reduced, a pattern shared among species in section *Corydalis* (Kim et al. 2023).

The genomic data generated in this study provide valuable resources for investigating phylogenetic relationships and refining the taxonomic classification of *Corydalis*. However, it should be noted that the phylogenetic reconstruction was based solely on chloroplast genomes, and the sampling of species was limited. Considering the genus’s high diversity and complex intraspecific variation, future studies will require broader taxon sampling and the integration of advanced sequencing approaches to fully resolve the evolutionary history of *Corydalis*.

## Data Availability

The data supporting the analysis and results of this study are openly available in Genbank under accession number PV658157 (http://www.ncbi.nlm.nih.gov/). The associated BioProject, SRA, and BioSample numbers are PRJNA1265137, SRR33639527, and SAMN48585436, respectively.
